# Counter Clinical Prognoses of Patients With Bloodstream Infections Between Causative *Acinetobacter baumannii* Clones ST191 and ST451 Belonging to the International Clonal Lineage II

**DOI:** 10.3389/fpubh.2019.00233

**Published:** 2019-08-16

**Authors:** Eun-Jeong Yoon, Dokyun Kim, Hyukmin Lee, Hye Sun Lee, Jong Hee Shin, Young Uh, Kyeong Seob Shin, Young Ah Kim, Yoon Soo Park, Jeong Hwan Shin, Seok Hoon Jeong

**Affiliations:** ^1^Department of Laboratory Medicine and Research Institute of Bacterial Resistance, Yonsei University College of Medicine, Seoul, South Korea; ^2^Biostatistics Collaboration Unit, Yonsei University College of Medicine, Seoul, South Korea; ^3^Department of Laboratory Medicine, Chonnam National University Medical School, Gwangju, South Korea; ^4^Department of Laboratory Medicine, Yonsei University Wonju College of Medicine, Wonju-si, South Korea; ^5^Department of Laboratory Medicine, Chungbuk National University College of Medicine, Cheongju-si, South Korea; ^6^Department of Laboratory Medicine, National Health Insurance Service Ilsan Hospital, Goyang-si, South Korea; ^7^Department of Internal Medicine, National Health Insurance Service Ilsan Hospital, Goyang-si, South Korea; ^8^Department of Laboratory Medicine and Paik Institute for Clinical Research, Inje University College of Medicine, Busan, South Korea

**Keywords:** *Acinetobacter baumannii*, bloodstream infection, international clonal lineage II, ST191, ST451

## Abstract

This study was conducted to evaluate the possible clinical and bacteriologic features associated with 30-day mortality from *Acinetobacter baumannii (A. baumannii)* bloodstream infections (BSIs). We conducted a prospective, multicenter, observational study of 181 entire episodes of *A. baumannii* BSI from six general hospitals between May 2016 and April 2017 in South Korea. Cox proportional-hazards regression model was used to estimate risks of the primary endpoint, i.e., all-cause mortality within 30 days from the initial blood culture. Most (84.5%) of the *A. baumannii* blood isolates belonged to the international clonal lineage II (ICLII) and 89.5% of the isolates were either multidrug- or extensively-drug resistant. We identified three risk factors including the old age of patient {hazard ratio, 1.033; [95% Confidential Interval (CI), 1.010–1.056]}, the sequential organ failure assessment score [1.133 (1.041–1.233)], and causative *A. baumannii* sequence type (ST) 191 belonging to ICLII [1.918 (1.073–3.430)], and three protective factors including causative *A. baumannii* ST451 belonging to ICLII [0.228 (0.078–0.672)], platelet count [0.996 (0.993–0.999)], and definitive therapy within 72 h [0.255 (0.125–0.519)]. Differing 30-day mortality rate in the dominant ICLII was observed by ST, which was much high in ST191 and low in ST451 and it was likely associated with the molecular traits, rather than the drug resistance.

## Introduction

*Acinetobacter baumannii* is dominant in clinical settings primarily targeting immunocompromised patients in intensive care units (ICUs) and has the potential to cause healthcare-associated infections (1). Infections caused by *A. baumannii* have repeatedly been reported to have grave clinical outcomes (2, 3). *A. baumannii* has an epidemic potential of emerging diseases because of its ability to persist in hospital environments by resisting desiccation and disinfectants (1). A propensity toward rapid acquisition of foreign DNA, including antimicrobial resistance determinants and virulence determinants, also contributes to its effective dissemination (4).

Previous studies have demonstrated that *A. baumannii* infections are accompanied by serious morbidity and mortality associated with a high sequential organ failure assessment (SOFA) score (5), comorbidities (6), immunosuppression (3), and antimicrobial treatment failure, mainly due to the multidrug resistance (MDR) of the causative *A. baumannii* (5, 7, 8). The rates of resistance to the major anti-*Acinetobacter* agents are increasing (9). In particular in South Korea, predominance of the multidrug-resistant international clonal lineage II (ICLII) in hospital settings is of great concern (10–12).

This study was performed to evaluate the possible clinical and bacteriologic features associated with 30-day mortality from *A. baumannii* bloodstream infection (BSI) for the entire 1-year incidence of *A. baumannii* BSI cases of multiple general hospitals in South Korea.

## Methods

### Study Design

A prospective, multicenter, observational study was designed to monitor the entire *A. baumannii* BSI episodes between May 2016 and April 2017 occurring in six general hospitals with 715–1,050 beds participating in a national surveillance study of antimicrobial resistance in South Korea (13). Local ethics committee approvals were expedited or waived at each participating hospital since the study had a purely observational nature. The *A. baumannii* BSI cases were detected through reviewing daily blood culture results. One authorized investigator at each hospital reviewed patients' medical records, all of the raw data were recorded in a pre-formatted spreadsheet, and the datasheet was submitted via email to an analysis center without monitoring. Prognoses of patients were followed for at least 30 days and the cases of transfer and hospital discharge were recorded as they were. The Charlson comorbidity index (14) and the SOFA score (15) were calculated in the analysis center using the collected raw data for the day of initial blood culture. For the sequential isolation of *A. baumannii* from one patient, the first isolate was used for analyses and the ensuing isolates were discarded. Among a total of 67,803 patients subjected to blood culture, *A. baumannii* isolates were recovered from blood specimens of 188 patients, and following the elimination of 7 episodes of death on the day of the initial blood culture, 181 cases were included in the analysis. The microbiological assessment was carried out in the analysis center.

### Definitions

Laboratory-confirmed *A. baumannii* BSI was defined as an *A. baumannii*-positive blood culture from one or more blood specimens (16). Hospital-originated (HO) infection was specified by ≥2 calendar days of hospitalization at the day of initial blood culture, including the time spent at a previous health care facility before transfer. The 30-day mortality was defined as all-cause mortality within 30 days from the initial blood culture. Non-susceptibility (NS) to a drug class was defined as NS to at least one drug in a class and resistance phenotypes were categorized following Magiorakos et al. (17): MDR (NS to three or more drug classes), extensively drug resistance (XDR, NS to at least one agent in all but one or two drug classes), and pan-drug resistance (NS to at least one agent in all drug classes). An empirical antimicrobial therapy was defined as an initial antimicrobial treatment before the susceptibility testing results for the causative pathogen. A definitive antimicrobial therapy was defined as prescribing a revised antimicrobial regimen based on the antimicrobial susceptibility testing results and, if the revised regimen was administered after 72 h, it was deemed to be a delayed definitive therapy. Adequate antimicrobial therapy must include at least one antimicrobial agent active *in vitro* and both the dosage and route of administration should meet current medical standards.

### Microbiological Assessment

Bacterial species were initially identified by matrix assisted laser desorption/ionization time-of-flight mass spectrometry using a Bruker Biotyper™ system (Billerica, MA, USA) and the detailed species of *Acinetobacter* spp. were confirmed by PCR and sequencing the *rpoB* gene. For *A. baumannii*, multilocus sequence typing was followed through the Oxford scheme (18). Antimicrobial susceptibility was tested following the Clinical and Laboratory Standards Institute (CLSI) guidelines (19). Antimicrobial susceptibilities to 13 anti-*Acinetobacter* agents belonging to seven drug classes were tested by the disk diffusion method. For carbapenem-resistant isolates, the *bla*_OXA−23_ gene and the IS*Aba1*-*bla*_OXA−51−like_ were identified by PCR and direct sequencing (20). Colistin MICs were determined by the broth microdilution method (21). Twenty-six virulence genes associated with eight virulence characteristics (22) were investigated by real-time PCR using gene-specific primers (23) and a KAPA SYBR Fast quantitative PCR master mix (Kapa biosystems, Inc., Wilmington, MA, USA). The resistance islands AbaR and AbGRI1 were specified by PCR using a set of primers, comMtr3_145-126F (5′-CAAGCGGTTGGCGTAAGACT-3′) and reverse primers tniB_546-525R (5′-CAAGTACATGCTCTGCAAGATG-3′) only for the full *tniB* gene and tniB_37-16R (5′-CAGACTCAATTCATTGCTGAGG-3′) for both the full and the truncated *tniB* genes.

### Statistical Analysis

Statistical analyses were performed in SPSS statistics (version 23, IBM Corp., Armonk, NY, USA). Data are expressed as the mean ± standard deviation if no special description is indicated. Categorical variables were compared by Pearson's X^2^ test, and continuous variables were compared by an independent two sample *t*-test. Associations between 30-day mortality and risk factors were evaluated using univariable and multivariable Cox proportional hazard regression models. The proportional hazards assumption was evaluated by including an interaction term between variables, and variables were natural-log transformed by follow-up time and by log-minus-log survival plots. For multivariable analyses, variables with a *P* value <0.1 in univariable analyses were considered to include any possible variables. Multicollinearity between variables was diagnosed by calculating the variance inflation factors, and a multivariable analysis was conducted by the backward stepwise method. All tests of significance were two-tailed; *P* values <0.05 were considered to be significant.

## Results

### Characteristics of Enrolled Patients

**Figure 1:**
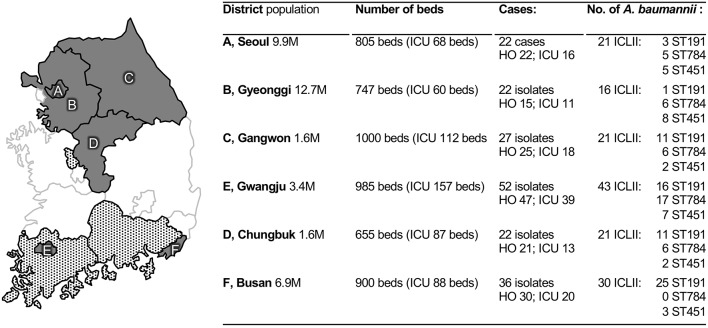
Participating sentinel hospitals and collected *Acinetobacter baumannii* blood isolates. Locations of the hospitals (in gray) and covering parts (in gray patterns) are indicated in a map of the South Korean peninsula, respectively, and the associated table represent information regarding blood isolates from each hospital including demographic information from 2016. HO, hospital-origin; ICLII, international clonal lineage II; ST, sequence type.

A total of 181 laboratory-confirmed *A. baumannii* BSI cases were enrolled from the six participating hospitals and the number of cases was ranged from 22 to 52 by hospital (Figure 1). Among patients with *A. baumannii* BSIs, the sex ratio of male *vs*. female was 115:66, and their mean age was 67.7 ± 14.3 years (Table 1). The mean Charlson comorbidity index score was 4.1 ± 2.2. Solid tumors (26.5%, 48/181), diabetes mellitus (23.2%, *n* = 42), cerebrovascular diseases (18.2%, *n* = 33), kidney diseases (13.8%, *n* = 25), and chronic pulmonary diseases (6.6%, *n* = 12) were the five most observed underlying diseases and dementia (*n* = 9) and chronic liver diseases (*n* = 7) were followed. Of the total, 88.4% (*n* = 160) of cases were HO infections, 95.0% (*n* = 172) were inpatients, and 68.0% of those (117/172) stayed in ICUs. The SOFA score had a mean value of 5.9 ± 3.5. Secondary BSIs were identified in 44.2% (80/181) of cases, and the majority (86.3%, 69/80) were originated from a pulmonary tract infection.

**Table 1:** Thirty day mortality-associated factors for patients with an *Acinetobacter baumannii* BSI.

**Variables**	Total^*^	**Survival**	**30-day death^†^**	**P^‡^**	**Univariate model**	
	***n* = 181**	***n* = 104**	***n* = 77**		**HR [95%CI]^§^**	***P***
**Demographic information**						
Male	115 (63.5%)	67 (64.4%)	48 (62.3%)	0.876	0.883 [0.557–1.400]	0.596
Age	67.7 ± 14.3	64.5 ± 15.5	72.1 ± 11.1	**<0.001**	1.033 [1.014–1.054]	<0.001
Inpatients	172 (95%)	98 (94.2%)	74 (96.1%)	0.735	1.355 [0.427–4.298]	0.606
Hospital origin	160 (88.4%)	87 (83.7%)	73 (94.8%)	0.033	2.808 [1.026–7.685]	0.044
Intensive care unit	117 (64.6%)	63 (60.6%)	54 (70.1%)	0.210	1.342 [0.824–2.187]	0.238
**Underlying disease**^¶^						
Charlson index	4.1 ± 2.2	3.9 ± 2.2	4.4 ± 2.2	0.108	1.086 [0.981–1.202]	0.112
Solid tumor	48 (26.5%)	27 (26.0%)	21 (27.3%)	0.866	1.183 [1.109–1.261]	<0.001
Diabetes mellitus	42 (23.2%)	28 (26.9%)	14 (18.2%)	0.213	0.049 [0.000–7177]	0.620
Cerebrovascular disease	33 (18.2%)	23 (22.1%)	10 (13.0%)	0.125	0.597 [0.307–1.161]	0.129
Kidney disease	25 (13.8%)	15 (14.4%)	10 (13.0%)	0.831	1.043 [0.631–1.722]	0.870
Chronic pulmonary diseases	12 (6.6%)	7 (6.7%)	5 (6.5%)	>0.999	6.176 [0.840–45.40]	0.074
Chronic liver diseases	7 (3.9%)	2 (1.9%)	5 (6.5%)	0.137	2.182 [0.878–5.420]	0.093
Dementia	9 (5.0%)	5 (4.8%)	4 (5.2%)	>0.999	1.069 [0.432–2.649]	0.885
Other^#^	5 (2.8%)	2 (1.9%)	3 (3.9%)	0.652	1.019 [0.372–2.787]	0.971
**Severity variables**						
SOFA score	5.9 ± 3.5	4.8 ± 3.2	7.4 ± 3.5	**<0.001**	1.183 [1.109–1.261]	<0.001
**Lab data**						
WBC	11.5 ± 7.8	12.2 ± 6.9	10.6 ± 8.8	0.185	0.967 [0.934–1.000]	0.052
Hb	10.4 ± 7.8	10.9 ± 10.4	9.6 ± 1.6	0.262	0.910 [0.788–1.051]	0.201
Platelet	169.7 ± 132.1	206.9 ± 138.3	119.5 ± 104.8	**<0.001**	0.994 [0.991–0.997]	<0.001
**Origin of infection^¶^**						
Pulmonary tract	69 (38.1%)	36 (34.6%)	33 (42.9%)	0.281	1.259 [0.801–1.978]	0.317
Wound	7 (3.9%)	5 (4.8%)	2 (2.6%)	0.700	0.653 [0.160–2.662]	0.553
Urinary tract	4 (2.2%)	4 (3.8%)	0 (0%)	0.138	0.048 [0.000–18.92]	0.319
**Causative pathogen**						
Polymicrobial infection	44 (24.3%)	30 (28.8%)	14 (18.2%)	0.116	0.636 [0.356–1.135]	0.125
ICLII^††^	153 (84.5%)	83 (79.8%)	70 (90.9%)	0.060	2.083 [0.958–4.533]	0.064
ST191 (1-3-3-2-2-94-3)	73 (40.3%)	29 (27.9%)	44 (57.1%)	**<0.001**	2.630 [1.671–4.139]	<0.001
ST784 (1-3-3-2-2-107-3)	31 (17.1%)	17 (16.3%)	14 (18.2%)	0.842	0.983 [0.551–1.755]	0.954
ST451 (1-3-3-2-2-142-3)	29 (16.0%)	24 (23.1%)	5 (6.5%)	0.004	0.293 [0.118–0.727]	0.008
**Virulence factors**						
Bacterial lipocalin, Blc	145 (80.1%)	79 (76.0%)	66 (85.7%)	0.132	1.540 [0.813–2.916]	0.185
Pro-apoptotic porin, PorB	146 (80.7%)	79 (76.0%)	67 (87.0%)	0.086	1.839 [0.946–3.575]	0.073
Small protein A, SmpA	177 (97.8%)	101 (97.1%)	76 (98.7%)	0.638	1.773 [0.247–12.75]	0.569
Pili, Csu	73 (40.3%)	45 (43.3%)	28 (36.4%)	0.363	0.781 [0.491–1.243]	0.297
PNAG, Pga	173 (95.6%)	96 (92.3%)	77 (100%)	0.022	22.02 [0.307–1579]	0.156
Polysaccharide exporter, EpsA	9 (5.0%)	7 (6.7%)	2 (2.6%)	0.305	0.425 [0.104–1.730]	0.232
Capsule protein, MviM	7 (3.9%)	4 (3.8%)	3 (3.9%)	>0.999	1.047 [0.330–3.322]	0.938
Lipopolysaccharide, VipA	16 (8.8%)	9 (8.7%)	7 (9.1%)	>0.999	1.021 [0.469–2.220]	0.959
Lipopolysaccharide, Wzx	48 (26.5%)	26 (25%)	22 (28.6%)	0.613	1.144 [0.698–1.875]	0.595
Glyconokinase, GntK	179 (98.9%)	102 (98.1%)	77 (100%)	0.508	20.54 [0.004–93770]	0.482
Quorum Sensing, LuxR	159 (87.8%)	91 (87.5%)	68 (88.3%)	>0.999	1.000 [0.499–2.004]	0.999
Motility and secretion, PilA	18 (9.9%)	12 (11.5%)	6 (7.8%)	0.460	0.723 [0.314–1.664]	0.446
Siderophore, BasD	179 (98.9%)	102 (98.1%)	77 (100%)	0.508	20.54 [0.004–93770]	0.482
**Antimicrobial resistance**						
Penicillins NS	157 (86.7%)	82 (78.8%)	75 (97.4%)	**<0.001**	7.352 [1.804–29.96]	0.005
3-, 4- gen. cephalosporins NS	162 (89.5%)	85 (81.7%)	77 (100%)	**<0.001**	25.33 [1.463–438.6]	0.026
Carbapenems NS	160 (88.4%)	84 (80.8%)	76 (98.7%)	**<0.001**	13.15 [1.828–94.63]	0.010
Aminoglycosides NS	141 (77.9%)	76 (73.1%)	65 (84.4%)	0.073	1.515 [0.818–2.805]	0.187
Fluoroquinolones NS	162 (89.5%)	85 (81.7%)	77 (100%)	**<0.001**	25.33 [1.463–438.6]	0.026
Tetracyclines NS	13 (7.2%)	11 (10.6%)	2 (2.6%)	0.045	0.284 [0.070–1.156]	0.079
Drug susceptible	18 (9.9%)	18 (17.3%)	0 (0%)	**<0.001**	0.040 [0.002–0.743]	0.031
MDR/XDR	162 (89.5%)	85 (81.7%)	77 (100%)	**<0.001**	25.33 [1.463–438.6]	0.026
**Treatment**						
Adequate empirical therapy	49 (27.1%)	30 (28.8%)	19 (24.7%)	0.613	0.744 [0.443–1.249]	0.263
Definitive therapy within 72 h	49 (27.1%)	39 (37.5%)	10 (13.0%)	**<0.001**	0.246 [0.124–0.488]	<0.001

* *The percentage was calculated from the total number of AB-BSI*.

† *All-cause mortality within 30 days from the initial blood culture*.

‡ *P value is either from Pearson's X^2^ test for categorical data or from the t-test for continuous data. Bonferroni corrected significance level from 32 hypotheses was considered to be P <0.0016, and the significant P values are indicated in bold*.

§ *Cox proportional hazards regression model was applied to calculate the hazard ratio (HR) and the 95% confidence interval (CI)*.

¶ *Underlying disease and primary site of infection could be multiple by patient*.

# *Ulcer (n = 2), connective tissue disease (n = 2), congestive cardiac insufficiency (n = 2), and leukemia (n = 2) are included*.

†† *Multilocus sequence typing profiles are indicated with the combination of alleles (gltA-gyrB-gdhB-recA-cpn60-gpi-rpoD)*.

*SD, standard deviation; ICLII, the international clonal lineage II*.

### The BSI-Causative *A. baumannii* Isolates

#### Sequence Types

The majority of the *A. baumannii* blood isolates belonged to ICLII (84.5%, 153/181) and the ICLII mostly comprised sequence type (ST) 191 (allele numbers of *gltA-gyrB-gdhB-recA-cpn60-gpi-rpoD*, 1-3-3-2-2-94-3; 47.7%, 73/153), ST784 (1-3-3-2-2-107-3; 20.3%, *n* = 31), and ST451 (1-3-3-2-2-142-3; 19.0%, *n* = 29). The 30-day mortality rate of patients with *A. baumannii* ICLII BSIs was 1.83-fold higher than that with *A. baumannii* BSIs by non-ICLII strains (45.8 vs. 25.0%); and among the ICLII BSIs, the 30-day mortality rate with ST191 BSIs was the highest and, the lowest for ST451 BSIs (60.3 vs. 17.2%).

#### Antimicrobial Resistance

Majority of the *A. baumannii* blood isolates (89.5%, 162/181) were either MDR or XDR, and the proportion was markedly greater in *A. baumannii* ICLII (98.7%, 151/153; 18 MDR and 133 XDR) than in non-ICLII (39.3%, 11/28; 5 MDR, and 6 XDR). All of the tested isolates were susceptible to colistin, and no pan-drug resistant isolate was observed. All but one carbapenem-NS *A. baumannii* harbored the *bla*_OXA−23_ gene (99.4%, 159/160) and the remaining one carried an insertion sequence IS*Aba1* upstream from the *bla*_OXA−51−like_ gene. Two thirds and one-quarter of *A. baumannii* possessed AbGRI1 (63.0%, 114/181) and AbaR (26.5%, 48/181), respectively.

#### Virulence Factors

Factors for adhesion and membrane integrity, biofilm formation, carbohydrate metabolism, quorum sensing, and siderophore were detected in *A. baumannii* blood isolates (Table 1). The acinetobactin-associated *basD* (98.9%, 179/181), the glycokinase *gntK* (98.9%, *n* = 179), the adhesion-associated *smpA* (97.8%, *n* = 177), and the biofilm formation-associated *pga* genes (95.6%, *n* = 173) were harbored by most of the *A. baumannii* blood isolates, while the capsule protein *mviM* (3.9%, *n* = 7), the polysaccharide exporter *epsA* (5.0%, *n* = 9), lipopolysaccharide *vipA* (8.8%, *n* = 16), and the type IV pili *pilA* (9.9%, *n* = 18) genes were occasionally identified. Among the virulence factors tested, the *pga* gene was significantly associated with 30-day death and the adhesion-associated *porB* had a tendency of 30-day mortality-associated.

A different frequency of each gene by ST was occasionally identified (Table 2). The *psaB* gene, formerly the *pab2* gene (24), was identified in all but one (96.6%, 28/29) ST451 isolates.

**Table 2:** Characteristics of the *A. baumannii* ICLII blood isolates.

**Characteristics**	**ICLII**	P^*^	**ST191**	P^*^	**ST451**	P^*^
	***n* = 153**		***n* = 73**		***n* = 29**	
Death within 30 days	70 (45.8%)	0.002	44 (60.3%)	0.000	5 (17.2%)	0.013
SOFA score (mean ± SD)	5.9 ± 3.6	0.005	6.2 ± 3.9	0.031	6.4 ± 3.6	0.161
Pulmonary tract of primary site of BSI	61 (39.9%)	0.000	24 (32.9%)	1.000	17 (58.6%)	0.002
**Drug resistance**						
MDR/XDR	151 (98.7%)	0.821	72 (98.6%)	0.003	29 (100%)	0.545
Penicillins NS	146 (95.4%)	0.000	70 (95.9%)	0.000	27 (93.1%)	0.004
3-, 4- gen. cephalosporins NS	151 (98.7%)	0.000	72 (98.6%)	0.000	29 (100%)	0.000
Carbapenems NS	149 (97.4%)	0.000	70 (95.9%)	0.000	29 (100%)	0.000
Aminoglycosides NS	136 (88.9%)	0.000	58 (79.5%)	0.006	29 (100%)	0.000
Fluoroquinolones NS	151 (98.7%)	0.000	72 (98.6%)	0.000	29 (100%)	0.000
Tetracyclines NS	11 (7.2%)	0.354	3 (4.1%)	0.553	6 (20.7%)	0.003
Polymyxin	0		0		0	
**VIRULENCE FACTORS**						
**Drug resistance**						
AbGRI1 (Tn*6022*)	112 (73.2%)	0.000	69 (94.5%)	0.000	5 (17.2%)	0.000
AbaR (Tn*6019*)	38 (24.8%)	0.164	2 (2.7%)	0.000	24 (82.8%)	0.000
OXA-23	148 (96.7%)	0.000	70 (95.9%)	0.000	28 (96.6%)	0.001
AdeC	32 (20.9%)	0.857	10 (13.7%)	0.110	6 (20.7%)	1.000
**Adhesion/Membrane Integrity**						
Blc	123 (80.4%)	0.002	56 (76.7%)	0.521	22 (75.9%)	1.000
PorB	124 (81%)	0.000	64 (87.7%)	0.001	23 (79.3%)	0.505
SmpA	149 (97.4%)	0.000	72 (98.6%)	0.002	27 (93.1%)	0.747
**Biofilm formation**						
Csu	133 (86.9%)	0.000	62 (84.9%)	0.000	29 (100%)	0.000
Pga	153 (100%)	0.000	73 (100%)	0.000	29 (100%)	0.002
**Capsule protein**						
EpsA	7 (4.6%)	0.742	1 (1.4%)	0.107	3 (10.3%)	0.160
MviM	6 (3.9%)	0.440	0 (0%)	0.099	2 (6.9%)	0.228
**Lipopolysaccharide**						
PsaB	36 (23.5%)	0.000	2 (2.7%)	0.000	28 (96.6%)	0.000
VipA	12 (7.8%)	0.448	7 (9.6%)	0.808	0 (0%)	0.084
Wzx	42 (27.5%)	0.129	17 (23.3%)	0.869	10 (34.5%)	0.172
**Carbohydrate metabolism**						
GntK	153 (100%)	0.000	73 (100%)	0.000	29 (100%)	0.003
**Quorum sensing system**						
LuxR	138 (90.2%)	0.000	65 (89%)	0.175	27 (93.1%)	0.183
**Motility and secretion**						
PilA	14 (9.2%)	0.808	2 (2.7%)	0.015	5 (17.2%)	0.164
**Siderophore**						
BasD	153 (100%)	0.000	73 (100%)	0.000	29 (100%)	0.010
**Treatment**						
Adequate empirical therapy	31 (20.3%)	0.000	16 (21.9%)	0.003	3 (10.3%)	0.002
Definitive therapy within 72 h	46 (30.1%)	0.013	20 (27.4%)	0.624	11 (37.9%)	0.109

* *P value is either from Pearson's X^2^ test for categorical data or from the t-test for continuous data. The Bonferroni corrected significance level from 33 hypotheses was considered to be P < 0.0015, and the significant P values are indicated with asterisks*.

#### Antimicrobial Treatment

The majority (95.0%, 172/181) of patients with *A. baumannii* BSIs received an empirical antimicrobial therapy with anti-*Acinetobacter* drugs and 28.5% (49/172) of those were adequate (Table 3). The other nine patients received either not-for-*Acinetobacter* drugs (*n* = 7) or none due to refusal of treatment (*n* = 2). Among the 132 patients who received an inadequate empirical therapy, 37.1% (*n* = 49) of patients were treated with a definitive antimicrobial therapy within 72 h and 20.4% (10/49) of those patients met death within 30 days, much less than that of patients who received a delayed definitive therapy (57.9%, 48/83).

**Table 3:** Antimicrobial regimens for the patients with *A. baumannii* BSIs.

**Regimen**	**No. of**	**cases**	**Proper**	**Improper**
Ampicillin	1	0.6%	1	0
Pipercillin/tazobactam	26	14.4%	5	21
comb.w/ciprofloxacin	12	6.6%	1	11
comb.w/tigecycline	2	1.1%	1	1
comb.w/colistin	2	1.1%	2	0
3-, 4- gen. cephalosporins	20	11.0%	5	15
comb.w/ciprofloxacin	8	4.4%	0	8
comb.w/aminoglycosides	2	1.1%	0	2
Carbapenems	55	30.4%	5	50
comb.w/aminoglycosides	2	1.1%	0	2
comb.w/co-trimoxazole	2	1.1%	1	1
comb.w/ciprofloxacin	9	5.0%	1	8
comb.w/colistin	10	5.5%	10	0
Tigecycline	6	3.3%	5	1
comb.w/co-trimoxazole	1	0.6%	1	0
comb.w/fluoroquinolones	2	1.1%	2	0
Colistin	4	2.2%	4	0
Ciprofloxacin	6	3.3%	4	2
Aminoglycosides	1	0.6%	0	1
Co-trimoxazole	1	0.6%	1	0
Not treated^*^	9	5.0%	0	9
	181		49	132

* *Patients received drugs not-for-Acinetobacter (n = 7) or none due to refusal of treatment (n = 2)*.

#### Risk Factors for 30-Day Mortality

Risk and protective factors associated with the 30-day mortality in patients with *A. baumannii* BSIs were searched for among the variables of *P* <0.1 through a Cox-regression univariable analysis. Most of the variables were omitted by stepwise selection procedure, and each three of risk factors and protective factors associated with the 30-day mortality in patients with *A. baumannii* BSIs were identified through a Cox-regression multivariable analysis (Table 4): the age of patients of hazard ratio (95% CI), 1.033 (1.010–1.056); the SOFA score of 1.133 (1.041–1.233); and the BSI-causative *A. baumannii* ST191 of 1.918 (1.073–3.430). Three protective factors were also identified: the platelet count of 0.996 (0.993–0.999), the BSI-causative *A. baumannii* ST451 of 0.228 (0.078–0.672), and the definitive therapy within 72 h of 0.255 (0.125–0.519).

**Table 4:** Risk factors associated with the 30-day mortality in patients with *A. baumannii* BSI.

**Variables**	VIF^*^	**HR [95%CI]^†^**	***P***
Age	1.058	1.033 [1.010–1.056]	0.005
SOFA score	1.335	1.133 [1.041–1.233]	0.004
Platelet	1.290	0.996 [0.993–0.999]	0.016
ST191	1.245	1.918 [1.073–3.430]	0.028
ST451	1.255	0.228 [0.078–0.672]	0.007
Definitive therapy within 72 h	1.094	0.255 [0.125–0.519]	<0.001

* *VIF, variance inflation factor. The collinearity was diagnosed for the variables including the multivariate analysis*.

† *A Cox-regression multivariate analysis was conducted by the backward method using the Wald model*.

## Discussion

During the surveillance study period, the overall incidence of *A. baumannii* was the third-highest (1.1/10,000 patient-days) BSI-causative Gram-negative bacteria followed by *Escherichia coli* and *Klebsiella pneumoniae*. Besides, the incidence was strikingly higher in ICUs (6.6/10,000 patient-days) than in general wards (0.4/10,000 patient-days) (25). The 30-day mortality rate of patients with *A. baumannii* BSIs is pretty varied by study depending on the subjected patients, and we observed 42.5% of 30-day mortality for entire *A. baumannii* BSI cases.

Among the variables studied, the old age and the high SOFA score were identified as the patient-associated risk factors. Those were reasonable hazardous factors since both factors pronounced the severity of illness directly or indirectly. A patient-associated protective factor, platelet counts in peripheral blood reflecting the patients' condition also made sense. As well, a treatment-associated protective factor, a proper definitive therapy within 72 h was comprehensible.

**Figure 2:**
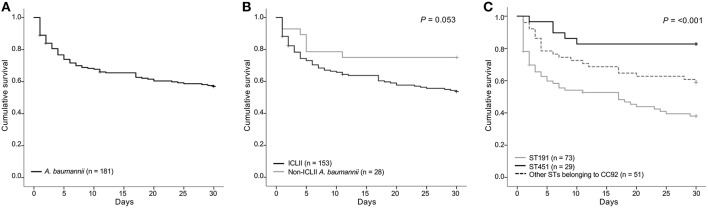
Mortality of BSI patients by causative pathogen. **(A)** Mortality of patients with BSIs by *A. baumannii*, **(B)** comparison of mortality of BSI patients by ICLII and by non-ICLII *A. baumannii*, **(C)** comparison of mortality of BSI patients by ST191, ST451, and other STs belonging to CC91. The Kaplan-Meier plot illustrated survival in patients with AB-BSI infected with the indicated bacterial clone. Differences between groups were compared by the Log-Rank test of the Mantel-Cox model, and the *P* values are presented. ICLII, international clonal lineage II; ST, sequence type.

Curiously among the causative-pathogen-associated variables, the *A. baumannii* ST191 was a risk factor, while the ST451, another ICLII member, was a protective factor. *A. baumannii* ICLII, is a well-known multidrug resistant clone (26, 27). The treatment options for patients infected by the clone are often limited to the very last resorts, such as carbapenems, tigecycline, minocycline, and polymyxins. For the infections cause by multidrug-resistant *A. baumannii*, the use of combination antimicrobial therapy is recommended (28) and two thirds (66.3%, 120/181) of the patients were treated empirically by antimicrobial combinations. Carbapenem resistance is already common in ICLII, and the remaining choices are always risky because tigecycline (29) and minocycline (30) have a bacteriostatic activity, which requires a combinational treatment; colistin has a severe dose-dependent neuro- and nephrotoxicity (31); and the colistin resistance is easily developed by prior use of the drug (32). The importance of an adequate antimicrobial therapy (33), including the proper use of colistin (34), has been addressed to diminish the 30-day mortality rate caused by *A. baumannii* infections. The present results confirmed the notion through a significant protective effect of the definitive therapy including colistin. Among ICLII members, ST191 was the most and both ST784 and ST451 are recently emerging ICLII clones (27, 32). In this study, ST784 and ST451 were the second and third most ICLII clones and the clones were evenly recovered from the six hospitals (Figure 2).

In this study, all but two (98.7%) ICLII blood isolates, one ST191 and the other ST357, exhibited MDR/XDR. Since drug resistance is one of the noted hazardous factors obstructing an adequate antimicrobial therapy (7, 8), similar mortality rates were presumed for BSIs caused by any STs belonging to the ICLII. However, a marked difference in 30-day mortality was observed in the three dominant STs: 60.3% in ST191 BSIs, 45.2% in ST784 BSIs, and 17.2% in ST451 BSIs. Curiously, the least proportion of patients with ST451 BSIs (10.3%) were treated by an adequate empirical therapy (Table 2), indicating that the drug resistance and the relevant adequacy of treatment were not related to the 30-day mortality rate at least to BSIs caused by ST191 and ST451. ST451 was more associated with a primary pulmonary tract infection (58.6%) than ST191 (32.9%), however the primary site of infection was insignificantly associated with a 30-day death (Table 1). Similarly, ST451 harbored the Tn*6019*-associated AbaR (82.8%) more frequently than the Tn*6022*-associated AbGRI1 (17.2%) differently from ST191 (2.7 vs. 94.5%), however no difference was expected since both may harbor the *bla*_OXA−23_ gene (12). The lipooligosaccharide biosynthesis-associated *psaB* gene was harbored strikingly more by ST451 than by ST191 (96.6 vs. 2.7%). The *psaB* gene is a part of the oligosaccharide gene clusters generating an ordinary side-branch sugar Gal, differing from the *A. baumannii* ST191 often carries the gene cluster producing FucNAc sugar composing the oligosaccharide (24). Since the lipooligosaccharides of pathogenic bacteria have long been recognized as a major virulence factor (35), the difference in oligosaccharides between the two clones is possibly associated with the 30-day mortality rate and the detailed impact should be further studied.

This study should be considered with the following caveats in mind. First, the enrolled cases had a lack of external validation due to a distinct epidemiology of *A. baumannii* clinical isolates in South Korea in terms of high carbapenem resistance, mostly conferred by OXA-23. Second, analyses of bacterial lipooligosaccharides differed by the clones are remained for further study. However, to the best of our knowledge, this is the first notification of the counter clinical prognoses of the patients with a BSI by dominant ICLII clones, drawing an attention to a further follow-up for the clonal exchange and expansion of ICLII clones in clinical settings.

This delicate prognosis assessment combined with an evaluation of the molecular epidemiology for the causative *A. baumannii* blood isolates confirmed (i) the predominance of ICLII in clinical settings, (ii) the hazardousness of antimicrobial resistance and the importance of an adequate antimicrobial therapy, and finally, (iii) we highlighted the differing 30-day mortality rates in patients with BSIs between causative multidrug-resistant *A. baumannii* clones ST191 and ST451 belonging ICLII probably due to the differing lipooligosaccharides.

## Data Availability

The raw data supporting the conclusions of this manuscript will be made available by the authors, without undue reservation, to any qualified researcher.

## Ethics Statement

The research, which has no involvement of human subject but the clinical isolates, does meet the exempt category without approval from Ethics Committee on Human Research of the Health Ministry in South Korea and the study design has not been reviewed by the committee.

## Author Contributions

SJ conceived the study. E-JY and SJ designed the study. E-JY, HSL, and SJ analyzed the data. E-JY and SJ wrote the manuscript. DK, HL, JongS, YU, KS, YK, YP, JeongS, and SJ collected the clinical data and bacterial isolates.

### Conflict of Interest Statement

The authors declare that the research was conducted in the absence of any commercial or financial relationships that could be construed as a potential conflict of interest.
